# COVID19 Pandemic and Physical Activity: An Observational Study on Sleep Quality and Anxiety

**DOI:** 10.3390/sports10030044

**Published:** 2022-03-16

**Authors:** Ausilia Elce, Aurora Daniele, Ilaria Loperto, Lucia De Coppi, Armando Sangiorgio, Angelina Vivona, Clorinda Sorrentino, Simona Iannaccone, Lucia Martiniello, Ersilia Nigro

**Affiliations:** 1 Dipartimento di Scienze Umanistiche, Università Telematica Pegaso, Isola F2, Via Porzio, 80143 Napoli, Italy; ausilia.elce@unipegaso.it (A.E.); ilaria.loperto@unipegaso.it (I.L.); luciadecoppi@gmail.com (L.D.C.); armando.sangiorgio@unipegaso.it (A.S.); angela.vivona@unipegaso.it (A.V.); clorinda.sorrentino@unipegaso.it (C.S.); simona.iannaccone@unipegaso.it (S.I.); lucia.martiniello@unipegaso.it (L.M.); 2 CEINGE, Biotecnologie Avanzate Scarl, 80131 Napoli, Italy; 3 Dipartimento di Medicina Molecolare e Biotecnologie Mediche, Università degli Studi di Napoli “Federico II”, 80131 Napoli, Italy; 4 Dipartimento di Scienze e Tecnologie Ambientali, Biologiche, Farmaceutiche, Università della Campania “Luigi Vanvitelli”, 81100 Caserta, Italy

**Keywords:** physical activity, COVID-19 pandemic, mental health, sleep, professional athletes

## Abstract

Mental alterations were described during the COVID-19 pandemic and sleep deprivation has been reported as a consequence of social isolation. In Italy, the COVID-19 pandemic spread out at the beginning of 2020 determining severe lockdown periods. The aim of our study was to observe the effects of lockdown on sleep and anxiety in trained non-professional subjects and professional athletes who continued to train during the lockdown period. Forty-six subjects (21 trained non-professional subjects and 25 professional athletes) were recruited from a variety of team and individual sports to complete a battery of previously validated and widely used questionnaires assessing psychometric and anthropometric parameters, physical activity levels, lifestyle habits, and sleep quality. Subjects were aged 27.0 ± 5.14. All items were evaluated as percentages and chi-square and Fisher’s exact tests were performed, as appropriate. Our data showed that the prevalence of the difficulty of falling asleep (over 30%), the tendency of nocturnal awakenings (over 30%), and moderate anxiety (over 38%) were at the same extent in the two groups. Of the professional athletes, 72.73% declared snoring during sleep *vs* 42.86% of non-professional subjects. No other significant differences were found between the two groups except for the perception of being constant in daily activity, significantly more reported by trained subjects (*p* < 0.005). Our data show a similar scenario of anxiety and sleep disturbances for the two groups, suggesting that lockdown by the COVID-19 pandemic has partially mitigated the known beneficial effects due to physical activity on mental health and sleep quality. Further analyses are necessary to define the associated risk factors.

## 1. Introduction

Sleep is a physiological reversible state in which unconsciousness occurs and it is characterized by regular intervals and loss of responsiveness to external stimuli, with generalized inactivity of muscles [1]. Sleep represents an essential and conserved function in mammals. The quality and quantity of sleep influence one’s health status from childhood [2] and are strictly associated with alterations of memory functions, cognitive performances, but also to metabolic and psychological alterations, weight gain, and obesity [3,4,5,6].

In adults, in the last three decades, sleep duration was reduced from 2 to 3 h per night. It has been recommended that adult sleep duration should be between seven and nine hours per night [7]. In addition, numerous studies of otherwise healthy individuals have shown poor sleep to be associated with poor emotional regulation [8], lower cognitive performance [3], and mood alterations [9]. Nevertheless, poor sleep, symptoms of depression, paresthesia, fatigue, and cognitive impairments have a negative impact on quality of life [10]. It follows, therefore, that their improvements should have a beneficial effect on the general quality of life [11].

Regular physical activity has a positive impact on fatigue, depression and, for example, cardiovascular fitness. Additionally, there is a growing body of research showing that exercise interventions can improve sleep [12]. Indeed, emerging evidence suggests that physical activity has a positive effect on sleep quality and mental health. Exercise has been found to be a modulator of depressive symptoms working better than other interventions such as meditation [13]. A recent meta-analysis about the effects of physical activity on depression and anxiety in adults showed that physical activity moderately decreases depressive symptoms as well as anxiety to a lower but significant extent [14]. In support of these data, two systematic reviews observed that physical activity is positively associated with emotional well-being and inversely with depression and anxiety [15,16,17]. In 2019, Ghrouz A.K. et al. observed a positive correlation between sleep, physical activity levels, and mental health among 617 college students aged between 18 and 30 years [18].

In athletes, sleep hygiene is strictly associated with performance, oxidative stress, and immune system health. Athletes are encouraged to put a high priority on their sleep pattern and, if necessary, to complement night’s sleep with a maximum of thirty-minute naps [18]. Few studies analyzed the effects of lockdown caused by the COVID-19 pandemic on athletes’ physical activity and sleep quality.

Italy was one of the first countries to adopt public health measures contrasting the rapid worldwide spread of COVID-19 by imposing a first severe lockdown from 9 March 2020 to 4 May 2020. During this period, the general population was forced to stay at home, going out only when strictly needed. Gyms and other sports structures were inaccessible to non-professional athletes. The second period of lockdown occurred at the beginning of 2021, and training was allowed only outside and individually, while the sports facilities continued to remain closed, with the exception of the facilities supporting competitive athletes’ training. It has been widely demonstrated that this unprecedented situation caused increased levels of anxiety, stress, and depression in the general population [19,20].

The aim of the present study was to observe the effects of the second lockdown period (from December 2020 to April 2021) on sleep quality and anxiety in 21 non-professional athletes compared to 25 professional athletes who continued to train during the lockdown period, for at least 5 weekly hours.

## 2. Materials and Methods

### 2.1. Subjects

A sample of 46 Italian subjects, from 18 to 35 years, comprising non-professional subjects (*n* = 21) who were not trained at the time of interview and professional athletes (*n* = 25) were recruited from Italy. Both groups were recruited in a volunteer manner from several sport professional associations in Campania Region, Italy. The participants resided in different regions of Italy, both from south and north. The sport associations took 3 months to recruit all subjects that were asked to fulfil the surveys within 2 months.

The mean age of participants was 27.0 ± 5.14 years.

The professional athletes trained for at least 5 weekly hours continuously during the lockdown; the non-professional subjects used to train 3 weekly hours before the lockdown but interrupted the activity. Athletes were excluded if they were: aged < 18 years, training and competing for <5 weekly hours, affected by sleep disorder, affected by metabolic syndrome, type 2 diabetes and over-weight/obesity. All subjects included in the study received an online synchronous consilience in which the aim of the study was described and an informative written consent to participate was collected. Following completion of the Supplementary Materials Survey S1, participants were informed about how to receive feedback from the survey.

### 2.2. Questionnaires

In the period from December 2020 to April 2021, we administered four semi-structured questionnaires through an online system. The first questionnaire consists of seven sections with structured questions alternated with open questions, the first section containing personal data (gender, date of birth, instruction, etc.) for a total of 12 questions, a second section regarding physical activity levels, lifestyle habits and anthropometric parameters that every subject had given through an at-home self-measurement, after receiving researcher instructions, for a total of 19 questions.

A psychometric section, for a total of 20 questions was set up to establish anxiety levels (mild anxiety, moderate anxiety, severe anxiety and panic level anxiety). The first survey included 14 questions about health state and health anamnesis with open questions, in order to collect pathological history, excluding subjects with metabolic syndrome, psychiatric disorders or obesity. A specific section on sleep quality was included for excluding subjects with overt sleep disturbances. After completing the first questionnaire, we administered three validated sleep questionnaires: Pittsburg Score Questionnaire standardized for Italians (PSQI) [21], Morningness Eveningness Questionnaire, (MEQ) [22], and Epworth Sleepiness Scale, (ESS) [23]. All questionnaires adopted in this study were administered via email with Google modules. In order to guarantee anonymity for the completed questionnaires, the subjects’ names were replaced by a numeric code. Google modules retrieve data from surveys in excel format. Graphs and figures were elaborated with excel.

### 2.3. Data Analysis

All data were analyzed using the Statistical SS^®^ 25.0 (IBM Corporation, Armonk, NY, USA). All items in the questionnaires were evaluated in percentages; chi-square and Fisher’s exact tests were performed, as appropriate.

For PSQI, the Shapiro Wilk test was used in order to test the distribution of the continuous variables. Since the distribution wasn’t normally distributed, non-parametric tests were performed. Results have been reported as number and percentage for categorical variables and as median and Inter Quartile Range (IQR) for continuous variables. In order to better show the differences between the two groups, PSQI single scores have been reported as mean and Standard Deviation. Mann–Whitney test, chi-square/Fisher’s Exact test has been used as appropriate. The multivariate logistic regression analysis has been performed considering latency as a dependent ordinal variable. The model was adjusted for sex, age, use of coffee, and sleep drugs. The multivariate regression analysis has been performed considering PSQI as dependent continuous variable. The model was adjusted for sex, age, occupation, use of coffee, energy drink, and sleep drugs. The level of significance was set at α ≤ 0.05.

## 3. Results

### 3.1. Participants’ Characteristics

In the first stage of the study, we recruited a total of 150 volunteers from sports clubs and sports associations. Unfortunately, only 46 of the 150 subjects completed the four proposed questionnaires. Forty-six subjects were included in this study: 25 of them professionals, who practiced the following sports disciplines also during lockdown: rugby (*n* = 15); weightlifting (*n* = 3); tennis (*n* = 2); CrossFit (*n* = 1); fitness (*n* = 4); and 21 non-professionals who practiced the following sports before lockdown: rugby (*n* = 1), weightlifting (*n* = 1), tennis (*n* = 1), running (*n* = 18) at non-professional levels. Our population was composed by 8 professional female athletes (height: 1.64 ± 0.66 m; weight: 58.08 ± 6.56 kg); 12 non-professional female athletes (height: 1.62 ± 0,11 m; weight: 59.63 ± 21.99 kg); 17 professional male athletes (height: 1.83 ± 0.13 m; weight: 94.01 ± 21.69 kg); and 9 non-professional male athletes (height: 1.75 ± 0.11 m; weight: 72.11 ± 18.59 kg). The Body Mass Index (BMI) of professional female athletes versus non-professional was 21.43 vs 22.56 kg/m^2^; BMI of professional male athletes versus non-professional was 27.91 vs 23.48 kg/m2. Of the professional male athletes, 76.47% played rugby, a sports specialty associated with elevated BMI values (Kind K. et al. 2015, [24]). The low number of samples between the categories “professional women”, “non-professional women”, “non-professional men” and “professional men” does not allow a statistical analysis and a comparison to be made on anthropometric parameters.

At the time of the interview, 88% of professional athletes were in a pre-competition training period, while 12% of them were at rest after a competition. Non-professional athletes were at rest due to lockdown restrictions. The general characteristics of the 46 subjects participating in the present study are summarized in Table 1. The two groups are comparable for all the analyzed features, except for sex.

### 3.2. Anxiety Level and Quality of Sleep 

Psychometric tests were administrated to all participants to establish anxiety level (mild, moderate, severe and panic level anxiety), evidencing that, at the same extent in the two groups, a moderate level of anxiety was recorded. In detail, 40% of professional athletes declared to feel tense and restless versus 39% of the non-professional athletes. Of the professional athletes, 35% reported to feel often in trouble, while 36% of the non-professionals had the same feeling at the interview time. Of the professional athletes, 25% felt they often had negative thoughts versus 28% of non-professional athletes.

Three questionnaires were administered to all participants to assess the duration and quality of sleep. The PSQI global score revealed that 44% of professionals and 23.81% of non-professionals had poor sleep quality (PSQI global score > 5) at the time of interview (*p* = ns).

The two groups are comparable for all the analyzed features, except for sex and previous alcohol or drug use. In particular, professional athletes show a statistically significant use of drugs and alcohol in the past with respect to non-professional athletes (*p* ≤ 0.05 Chi-squared test/Fisher Exact Test). The two populations show no differences, except for the parameter linked to the latency of falling asleep (Table 2). Professional athletes seem to take longer to fall asleep, however, correcting the univariate and considering the latency parameter as dependent in a multivariate logistics corrected for sex, age, use of coffee, and sleep drugs, the parameter is not significant between the two groups (data not shown).

In Table 3, multivariate regression analysis was reported for the PSQI scores. A correlation emerges between high PSQI values (which correspond to low quality of sleep) and the use of drugs for sleep (*p* = 0.028).

**Figure 1:**
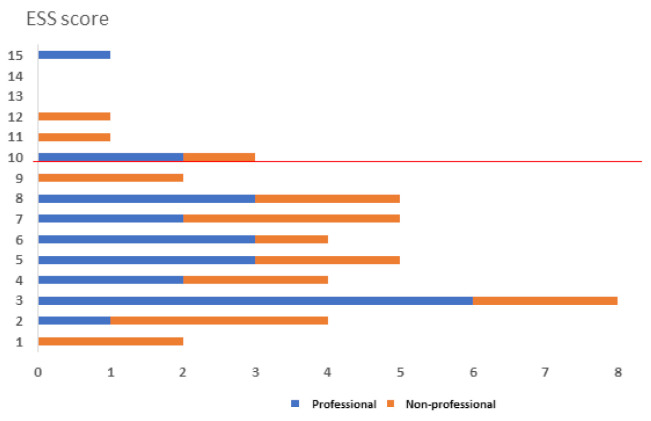
ESS scores are reported for professionals (blue bars) and non-professional athletes (orange bars). Scores >10 indicate a tendency to excessive sleepiness.

In addition, participants completed the Epworth Sleepiness Scale (ESS) as a measure of general sleepiness, where a score of >10 indicates excessive sleepiness (Johns, 1991), a score >12 indicates subjects with mild obstructive sleep apnea (OSA) and a score > 12 indicates a severe OSA [23]. Scores collected from the two groups are reported in Figure 1. For both groups, 13.5% of subjects show an ESS score >10 that is correlated with an increased tendency of daytime sleepiness.

**Figure 2:**
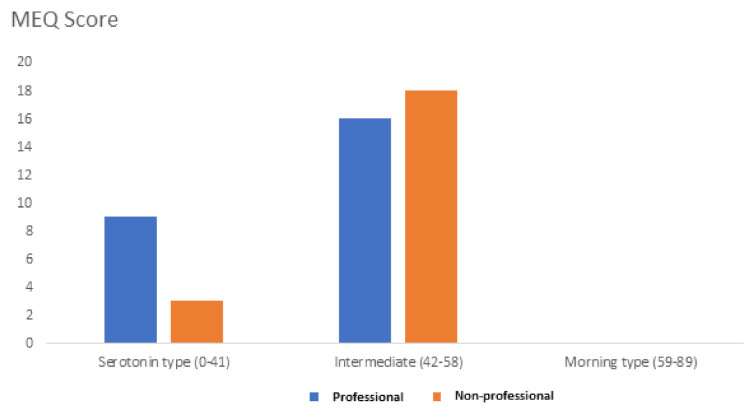
MEQ score was obtained from professionals (blue bars) and non-professionals (orange bars).

Finally, we administered the MorningnessEveningness Questionnaire (MEQ) in order to evaluate the circadian rhythm of each subject in relation to the peak of alertness in the morning, in the evening, or in between. The standard MEQ consists of 19 multiple-choice questions with the scores falling in a range between 16 and 86. A score lower or equal to 41 indicates a “serotonin” type. A score greater than or equal to 59 indicates a “morning” type. Scores between 42 and 58 indicate an “intermediate” type. The 36% of professionals versus the 14% of non-professional subjects reached a score comprised between 0 and 41, which corresponds to a “serotonin type” (Figure 2).

We found that the prevalence of difficulties to sleep was about 44% in the professional group versus 23% in non-professionals. Interestingly, 40% of professionals and 38% of non-professionals reported a tendency to wake up during sleep and 32% of professionals reported feeling often tense and restless versus the 14% of non-professionals. 

## 4. Discussion

Exercise and sports activities were demonstrated to positively impact behavioral sleep parameters [25]. Indeed, exercise may impact both the need for sleep and the circadian pacemaker. However, it has been shown that the effects are produced for long-term training and are not determined by single bouts of exercise [26].

Our data analysis from 46 subjects included in this study revealed no statistically significant differences in terms of sleep quality and duration between non-professional subjects, who interrupted physical activity during the severe lockdown, and professional athletes, who continued with a training volume of at least 5 weekly hours. Surprisingly, professional athletes show a tendency of the latency of falling asleep more prolonged than non-professional athletes, despite training.

The absence of relevant differences between the two groups might be attributable to several factors. Firstly, the non-professional subjects considered in this study were physically active until the beginning of the lockdown and potentially still benefit from the positive effects of exercise on sleep parameters.

Secondly, a prolonged latency to fall asleep was observed in young athletes (16–33 years) and is correlated to the use of devices in the 2 h before going to bed. Our professionals declared to use devices like smartphones and tablets for a mean of 2 ± 1.48 h before sleeping, while non-professionals used the devices for 1.2 ± 0.99 h. Knufinke M. et al. [27] registered that about 70% of elite athletes used electronic devices before sleeping. It has been shown that 2 h of exposure to an electronic tablet suppress melatonin levels by 23% (which would be expected to subsequently delay sleep onset), but melatonin levels are not significantly suppressed after only 1 h of device use [28]. The effect seems to be due to short-wavelength light emitted from electronic device screens that can suppress the nocturnal increase in melatonin; other studies showed that the latency of falling asleep is correlated to the cognitive load of devices utilization before sleep [29]. In the present study, the mean sleep duration reported was 7.5 ± 1.10 h per night.

In addition, it is to notice that self-reported data are not as accurate as more objective measures of sleep, such as laboratory-based polysomnography, or actigraphy, particularly with regard to measures related to the latency of falling asleep and waking after sleep onset [30,31]. Caia J. et al. (2018) [31] compared sleep duration measured with actigraphy and sleep diaries in rugby players and, although highly correlated (r = 0.85), the self-reported sleep duration was overestimated by approximately 20 min. In accordance, some studies have also shown that athletes overestimated sleep duration of 58 ± 85 min and underestimated time of sleep onset of 37 ± 72 min with questionnaires [32].

The prevalence of anxiety, sleep disturbance, and negative perceptions found in the athletes of this study during the second wave of the COVID-19 pandemic is in accordance with other studies that found a high prevalence of negative habits and lifestyle changes in athletes due to isolation [33]. Confinement at home can lead to poor and inappropriate nutrition, poor quality of sleep, addictions, loneliness, and negative lifestyle changes. The physiological adverse effects of isolation include an increase in body fat content and a decrease in muscle mass, impaired immunity, loss of mental sharpness and toughness, insomnia, and depression, as reported by Chen P. et al., 2020 [34], and Halabchi F. et al., 2021 [35].

Surprisingly, our data show a similar scenario of anxiety and sleep disturbances between the two groups of professional and non-professional athletes, suggesting that lockdown caused by the COVID-19 pandemic has partially mitigated the known beneficial effects due to the physical activity on mental health in both trained subjects and professional athletes, leading to the same mood and sleep disturbances of untrained subjects. This observation underlies the need of implementation plans to improve studies on anxiety disorders considering that anxiety and depression lead to negative effects on various quality of life domains. Regular and structured physical activity is a factor that improves several health parameters. However, elite sports specialties have peculiar stress-genic factors related to competition that could impact on mental health, perturbing sleep and anxiety levels. We hypothesized that these stress-genic factors are associated with the lack of sociability and by the uncertainty caused by severe restrictions imposed by the COVID-19 pandemic introduced in the period of our study. In this study, we focused on mental well-being, in particular during self-isolation and social distancing policies linked to the COVID-19 pandemic. However, our data underlie the need of further studies to assess how different intensities of physical activities may improve mental well-being during the COVID-19 pandemic.

Nonetheless, these results must be interpreted with prudence as a limitation of the study should be borne from the relatively small number of recruited subjects and from the number of the subjects belonging to each group (sports specialty) that did not reach statistical significance, but it can give an indication of stressor phenomena determined by severe lockdown in trained subjects.

Further analysis, using appropriate scores and an increased population study, is necessary to better define the phenomenon and the associated risk factors linked to severe lockdown. The effects of the limitations to sociability linked to training and competitions due to the COVID-19 pandemic remain to be investigated. However, it seems urgent to draw up rules and suggestions to face possible future lockdown periods. Tayech A. et al. defined best practices for athletes in order to maintain healthy habits during isolation. Among these: to cure adequate sleep hygiene and sleep routine, avoid eating about 3–4 h before sleeping, avoid caffeine or other stimulating drinks about 4–5 h before sleep, avoid the overuse of social networks (Twitter, Facebook, Instagram) and electronic devices (smartphone, tablet, etc.) in the late-night [32].

## Figures and Tables

**Table 1:** Main features of subjects participating in the study.

	Non-Professionals	Professionals	*p*-Value *
*n*, %	21 (45.65)	25 (54.35)	-
Sex, M (*n*, %)	8 (32)	17 (68)	***0.043***
Age Class (*n*, %)			
			0.731
10–19	3 (42.86)	4 (57.14)	
20–29	9 (42.86)	12 (57.14)	
30–39	9 (52.94)	8 (47.06)	
Occupation (*n*, %)			
Student	8 (40.00)	12 (60.00)	0.632
Unemployed	3 (60.00)	2 (40.00)	
Employed	9 (52.94)	8 (47.06)	
Employed in sports	1 (25.00)	3 (75.00)	
Education Attainment (*n*, %)			
Middle School	0 (0.00)	1 (100)	0.716
High School	12 (42.86)	16 (57.14)	
First level degree	5 (55.56)	4 (44.44)	
Second level degree	4 (50.00)	4 (50.00)	
Pathological history (*n*, %)			
Sleep disorders	3 (33.33)	6 (66.67)	0.408
Cups of coffee a day (*n*, %)			
0	2 (22.22)	7 (77.78)	0.118
1	17 (56.67)	13 (43.33)	
2	2 (28.57)	5 (71.43)	
Energy Drink, yes (*n*, %)	5 (50.00)	5 (50.00)	0.755
**Use of sleep medications or supplements (*n*, %)**	1 (16.67)	5 (83.33)	0.141

* Chi squared test/Fisher Exact Test.

**Table 2:** PSQI scores in 21 non-professionals and in 25 professional athletes.

	Non-Professionals (*n*: 21)	Professionals (*n*: 25)	*p*-Value
**PSQI (median, IQR)**	3 (2–3)	5 (4–5)	0.125 *
**PSQI (*n*, %)**			
Poor sleep quality	5 (31.25)	11 (68.65)	0.152 **
Good sleep quality	16 (53.33)	14 (46.67)	
**PSQI Single Scores (mean, ± sd)**			
duration of sleep ***	0.43, ±0.81	0.28, ±0.54	0.6560 *
sleep disturbance ***	1.00, ±0.45	1.08, ±0.57	0.5859 *
sleep latency ***	1.00, ±01.05	1.56, ±0.87	0.0340 *
day dysfunction due to sleepiness ***	1.00, ±0.63	1.16, ±0.62	0.2470 *
sleep efficiency ***	0.62, ±0.86	0.56, ±0.58	0.8338 *
overall sleep quality ***	0.19, ±0.51	0.32, ±0.80	0.7912 *
need meds to sleep ***	0.14, ±0.35	0.08, ±0.28	0.4998 *
* Mann–Whitney Test			
** Chi-Squared/Fisher Exacta Test			

* Mann-Whitney Test. ** Chi Squared/Fisher Exacta Test. *** Minimum Score = 0 (better); Maximum Score = 3 (worse). Shapiro Wilk’s test was used to test the normal distribution of the PSQI variable. The distribution is not normal so median, IQR, and non-parametric tests are reported.

**Table 3:** Multivariate regression analysis for PSQI score and other variables.

	Coef.	Std. Err.	*t*	*p*	[95% Conf.	Interval]
**Sex, F (ref: M)**	0.217	1.144	0.190	0.851	−2.114	2.548
**Age**	−0.028	0.099	−0.280	0.779	−0.230	0.174
**Occupation, ref: Student**						
Unemployed	1.626	1.758	0.920	0.362	−1.955	5.207
Employed	1.153	1.244	0.930	0.361	−1.381	3.688
Employed in sports	0.843	1.836	0.460	0.649	−2.896	4.583
**Cups of coffee a day, ref: 0**						
1	−2.248	1.384	−1.620	0.114	−5.067	0.571
2	−1.116	1.509	−0.740	0.465	−4.190	1.958
**Energy Drink, ref: no**	0.416	1.072	0.390	0.700	−1.767	2.599
**Past alcohol or drug use. ref: never**						
Yes. Seldom	0.432	1.464	0.300	0.770	−2.550	3.415
Yes. Often	1.270	1.776	0.720	0.480	−2.347	4.887
**Use of sleep medications or supplements; ref: no**	3.529	1.534	2.300	0.028 *	0.403	6.654
**Sport Category, ref: Sedentary**	0.454	1.131	−0.400	0.691	−2.758	1.850
Multivariate regression; Dependent variable: PSQI (continuous)						

* Mann-Whitney Test.

## Data Availability

Not applicable.

## Supplementary Materials

The following are available online at https://www.mdpi.com/article/10.3390/sports10030044/s1, Survey S1.
