# Supplementary material for: COVID19 Pandemic and Physical Activity: An Observational Study on Sleep Quality and Anxiety
Source: Sports (Basel). 2022 Mar 16;10(3):44. doi: 10.3390/sports10030044 (PMC8948688; doi:10.3390/sports10030044)
Supplement: Supplementary file 1 [file sports-10-00044-s001.zip › sports-1574503-supplementary.pdf]

Survey n°1

Section 1: Anamnestic section

1. Name and surname
   Please, insert your name and surname
2. Date of birth
3. Place of birth
   Please, indicate here the municipality and the province of birth, for example, Cecina (Livorno)
4. Gender
5. Is it currently in quarantine due to COVID-19? Yes/No
6. Phone number
   Please, indicate here your telephone contact (e.g. mobile number) that we will use exclusively to provide you with further information and clarifications relating to the study.
7. Email contact
   Enter your email contact here (e.g. mario.rossi@gmail.com) which we will use to provide you with electronic material relating to the study (e.g. informed consent form, instructions for participation and description of the study, any results).
8. Actual employment
   Please indicate your current main occupation below. If you are a university student, please also enter the faculty, for example, Student (Law).
9. Educational qualification.
   Please enter the highest academic qualification you have achieved here.
   Specify here if, after obtaining a degree, he continued his studies without finishing the cycle (e.g. after middle school, three years of high school).
10. Please indicate on this scale your motivation to take part in the experiment, from 1 to 5.
11. Have you already read the information text and signed the informed consent? Yes/No/Not yet
12. Have you understood the objectives of the study?

Section 2: Breastfeeding and pregnancy

1. Are you breastfeeding? Yes/No

2. Are you pregnant? Yes/No

Section 3: Anamnestic information. Please specify your health status.

1. Did you have any specific learning problems? Specific learning disorders are conditions found in school-age children, characterized by difficulty or delay in learning some specific skills (such as writing or reading), although the individual's intelligence is normal.
2. Did you have problems with mental and intellectual development? These kinds of disorders are generally disabling, they mainly have an onset in childhood and adolescence but affect the whole life span.
   - Pervasive developmental disorder (e.g. autism, Asperger's syndrome, Rett's syndrome)
   - Schizophrenia and Psychosis
   - Mental delay
   - Attention-Deficit / Hyperactivity Disorder (ADHD)
   - Conduct disturbance (violation of the company's rules of conduct)
   - No, I have not had any problems
   - Other, specify

3. Do you suffer from any chronic pathology? In this case, a chronic disease is understood to mean a disease that has symptoms that are constant over time and generally stable, which requires regular pharmacological treatment (e.g. asthma, ulcerative colitis, lupus erythematosus, psoriasis, metabolic syndrome, diabetes) Yes/No, please, if Yes specify.
4. Are you currently taking any medications? Yes/No, please, if yes specify.

Section 4: Additional medical informations

1. Do you suffer from or have you suffered in the past from the following disease? Please read the list carefully and indicate if one or more of the following conditions apply to you. Otherwise, check the option "No, I do not suffer nor have I ever suffered from a particular disease"
    - Respiratory disorders (e.g. asthma, dyspnoea, pneumonia, cystic fibrosis)
    - Cardiac/circulatory disorders (e.g. arrhythmia, hypertension, heart failure, congenital heart disease, valvular and vascular disorders, cardiomyopathy)
    - Endocrinological disorders (e.g. thyroid disease, adrenal gland disease, diabetes mellitus, polycystic ovary)
    - Renal, hepatic and gastrointestinal disorders (e.g. renal failure, hepatitis, Crohn's disease, celiac disease)
    - Neurological and neuromuscular diseases (e.g. epilepsy, stroke, headache, migraine, Alzheimer's disease, Parkinson's disease, dystrophy, multiple sclerosis, myopathy)
    - Psychiatric disorders (e.g. anxiety disorder, depression, psychosis, obsessive-compulsive disorder, eating behaviour disorder)
    - Sleep disturbances (e.g. insomnia, hypersomnia, narcolepsy, sleepwalking, bruxism, sleep apnea, restless legs syndrome)
    - Oncological disorders (e.g. leukaemia, lymphoma, melanoma)
    - No, I do not suffer nor have I ever suffered from any particular disease
    - Other
2. Have you ever lost consciousness? Yes/No
3. Have you ever abused alcohol and/or drugs?
    - Yes, frequent abuse of alcohol and/or drugs
    - Yes, I have frequently abused alcohol and/or drugs in the past
    - Yes, sometimes I happen to abuse alcohol and/or drugs
    - Yes, in the past I have occasionally abused alcohol and/or drugs
    - No, I have never abused alcohol and/or drugs
4. How much coffee do you drink per day?
    - 0 Cup a day
    - 1-3 Cups a day
    - 4-6 Cups a day
    - More than 6 Cups a day
5. Do you use other stimulating / energizing drinks daily? (e.g. ginseng; tea; redbull)? Yes/No
6. How do you rate your health over the past two weeks? Please, indicate a motivation scale from 1 to 5

    Section 5: Psychometric Questionnaire. Below are some phrases that people use to describe themselves. Read each sentence and choose from the options how you usually feel. There are no right or wrong answers and you don't take too long to answer. Give the answer that seems to best describe how you usually feel

1. I feel good
    - Almost never
    - Sometimes

- Often
- Almost always

2. I feel tense and restless
    - Almost never
    - Sometimes
    - Often
    - Almost always
3. I am satisfied with myself
    - Almost never
    - Sometimes
    - Often
    - Almost always
4. I wish I could be as happy as others seem
    - Almost never
    - Sometimes
    - Often
    - Almost always
5. I feel like a failure
    - Almost never
    - Sometimes
    - Often
    - Almost always
6. I feel rested
    - Almost never
    - Sometimes
    - Often
    - Almost always
7. I am calm, peaceful and in control of myself
    - Almost never
    - Sometimes
    - Often
    - Almost always
8. I feel that the difficulties accumulate so much that I cannot overcome them
    - Almost never
    - Sometimes
    - Often
    - Almost always
9. I worry too much about things that don't really matter
    - Almost never
    - Sometimes
    - Often
    - Almost always
10. I’m happy
    - Almost never

- Sometimes
- Often
- Almost always

11. I get negative thoughts
    - Almost never
    - Sometimes
    - Often
    - Almost always
12. I lack confidence in myself
    - Almost never
    - Sometimes
    - Often
    - Almost always
13. I feel safe
    - Almost never
    - Sometimes
    - Often
    - Almost always
14. I make decisions easily
    - Almost never
    - Sometimes
    - Often
    - Almost always
15. I feel inadequate
    - Almost never
    - Sometimes
    - Often
    - Almost always

16. Thoughts of little importance go through my mind and annoy me

    - Almost never
    - Sometimes
    - Often
    - Almost always
17. I live the disappointments with such participation that I cannot get them out of my head
    - Almost never
    - Sometimes
    - Often
    - Almost always
18. I am a constant person
    - Almost never
    - Sometimes
    - Often
    - Almost always
19. I get tense and upset when I think about my current concerns
    - Almost never
    - Sometimes

- Often
- Almost always

20. I feel satisfied with my life
    - Almost never
    - Sometimes
    - Often
    - Almost always

Section 6: Sleep quality Questionnaire.

1. Do you have difficulty falling asleep? Yes/No
2. Do you tend to wake up very early in the morning? Yes/No
3. Do you tend to wake up during the night? Yes/No
4. Have you ever been told that you snore during your sleep? Yes/No
5. Have you ever been told that you grind your teeth while you sleep? Yes/No

Section 7: Physical activity levels and anthropometric questionnaire

1. Please indicate your current weight measured in the morning, without clothes.
2. Please indicate your current height in meters.
3. Were you breastfed as a newborn? Yes/No
4. Are you right-handed? Yes/No
5. Enter the circumference of the right wrist in centimetres
6. Enter the circumference of the left wrist in centimetres
7. Please indicate your waist circumference in centimetres
8. Indicate the hips circumference in centimetres (measuring the widest part of the pelvis with a tape measure)
9. Indicate the abdomen circumference in centimetres (measuring the most prominent part of the abdomen with a tape measure)
10. Indicate the umbilical circumference in centimetres (measuring the circumference at the navel with a tape measure)
11. How many hours a day are you sitting?
12. How many hours a day are you watching or playing video games?
13. Do you use food supplements? Yes/No
14. Please specify if Yes
15. Which sport does it currently play?
16. Are you currently training? Yes/No
17. How many hours do you train a week?
    - More than 5 hours a week
    - Less than 5 hours a week
18. Do you run competitions on a national level?
19. At the date of compiling this questionnaire you are in the phase of:
    - pre-competition training
    - play today / have played a match today
    - rest after the race
    - I don't train
